# Post-transfusion severe headache in a patient with thalassemia with superficial siderosis of the central nervous system: a case report and literature review

**DOI:** 10.1186/s12883-024-03526-1

**Published:** 2024-01-06

**Authors:** Xudong Liu, Hongliang Jiang, Lijie Ren, Liming Cao

**Affiliations:** 1 Department of Neurology, The First Affiliated Hospital of Shenzhen University, Shenzhen, China; 2 Department of Neurology, The Third People’s Hospital of Yiyang City, Yiyang, China; 3 Department of Neurology, Shenzhen Second Peoples Hospital, Shenzhen, China; 4 Hunan Provincial Key Laboratory of the Research and Development of Novel Pharmaceutical Preparations, Changsha Medical University, Changsha, China

**Keywords:** Severe headache, Blood transfusion, Thalassemia, Superficial siderosis of the central nervous system, Case report

## Abstract

**Background:**

Patients with severe thalassemia may experience adverse effects from transfusion such as fever, rash, and iron overload after long-term transfusion therapy. Severe headaches as a side effect of blood transfusion in patients with thalassemia are not commonly observed, especially when combined with superficial siderosis of the central nervous system, which is easily misdiagnosed and requires excessive examination and treatment.

**Case Presentation:**

A 31-year-old woman was admitted with severe headache and vomiting over 3 days following blood transfusion. She was diagnosed with intermediate α-thalassemia at 2 years of age and had a history of irregular blood transfusions. Physical examination revealed horizontal nystagmus with no other abnormal neurological signs. Magnetic resonance (MR) imaging, MR venography, MR arteriography, and cerebrospinal fluid analysis were normal. However, susceptibility-weighted imaging showed abnormal signals in the bilateral and fourth ventricles. Initial antibiotics, antivirals, decompression of intracranial pressure, iron chelation, and symptomatic treatments were administered; subsequently, small intermittent blood transfusions were cautiously administered for severe anemia. The patient’s headache was gradually relieved, and she was discharged on day 9. At the 5-month follow-up, the patient’s headache recurred following another transfusion.

**Conclusions:**

Severe post-transfusion headache in patients with thalassemia has not been fully recognized and is easily misdiagnosed, leading to excessive examination and treatment. Understanding the clinical features of transfusion-related headaches can help identify this complication, but the exact pathophysiological mechanism requires further research.

## Background

Thalassemia is a hemolytic anemia in which normal adult-type hemoglobin synthesis is reduced owing to a defect in one or more peptide-chain genes, leading to impaired biosynthesis of the peptide chain [1]. Thalassemia is a hereditary hematological disorder caused by gene mutations or deletions, resulting in disorders of globin synthesis. Thalassemia can be divided into α and β types according to different peptide-chain synthesis disorders and further divided into mild, intermediate, and severe [1, 2]. The primary characteristic of thalassemia is the impaired production of hemoglobin, leading to a decrease in the number of red blood cells (RBCs), abnormally shaped RBCs, and shortened RBC lifespan, resulting in anemia. Due to the extensive destruction of RBCs, patients with Mediterranean anemia often suffer from severe anemia. RBC transfusion therapy is often required to correct anemia. After receiving RBC transfusion, certain patients may experience headaches, possibly associated with physiological changes during the transfusion process. However, there is insufficient comprehensive data and understanding of the related mechanisms. Severe headache as a post-transfusion complication in patients with thalassemia is not well known to clinicians, especially when combined with cerebral superficial siderosis (CSS), which is easily misdiagnosed in patients with thalassemia.

Hemoglobin disorders present a significant health problem in 71% of 229 countries, with this 71% including 89% of all births worldwide. Over 330,000 infants born annually are affected, with 17% affected by thalassemia [3]. Thalassemia is mainly concentrated in tropical and subtropical regions and is prevalent on the Mediterranean coast, North Africa, Southeast Asia, and the Indian subcontinent. The incidence of thalassemia is also high in southwestern and southern China [4]. Blood transfusion is an important treatment for severe thalassemia. Blood transfusion-related central nervous system (CNS) complications (e.g., headache, cognitive impairment, and cerebral hemorrhage [5–7]) are rare in patients with thalassemia and must be distinguished from migraine, intracranial infections, and intracranial venous sinus thrombosis. To the best of our knowledge, there have been no reports of patients with thalassemia with CSS developing severe headaches after transfusion.

Here, we present a case report demonstrating this scenario. We outline the implications of the case study, summarize the clinical characteristics, and analyze the possible mechanisms of transfusion-related CNS complications to improve our understanding and management of rare transfusion-related neurological complications.

## Case presentation

**Fig. 1:**
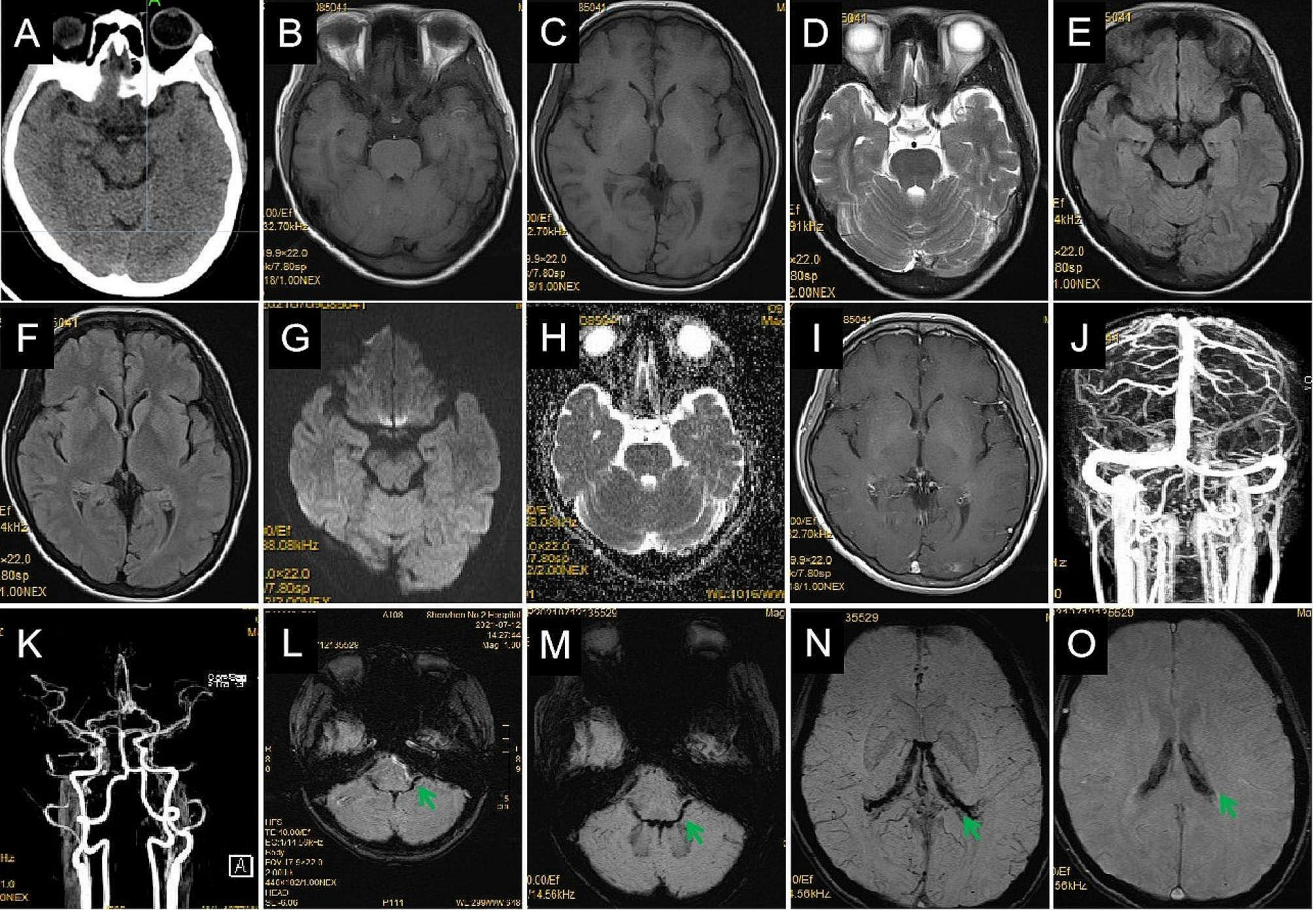
The patient’s CT and MR imaging findings. The initial brain CT (**A**), brain MR imaging (**B** and **C**, T1-WI; **D**, T2-WI; **E** and **F**, fluid-attenuated inversion recovery sequences; **G**, diffusion-WI; **H**, apparent diffusion coefficient), enhanced MR imaging (**I**), MR venography (**J**), and MR arteriography (**K**) show no obvious abnormalities. Susceptibility-weighted imaging (**L**-**O**) shows abnormal signals in bilateral ventricles, the fourth ventricle, and the brainstem surface. CT, computed tomography; MR, magnetic resonance; WI, weighted imaging

**Fig. 2:**
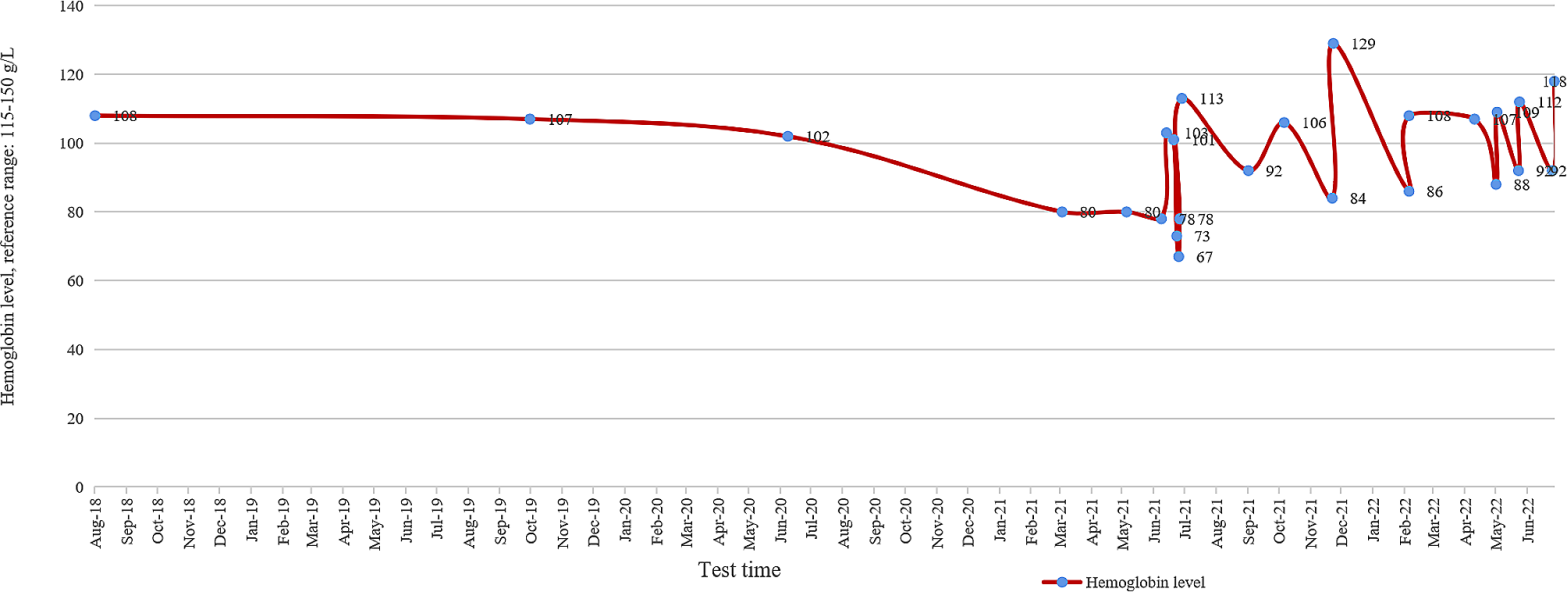
Trend line of the patient’s hemoglobin level from August 2018 to June 2022

**Fig. 3:**
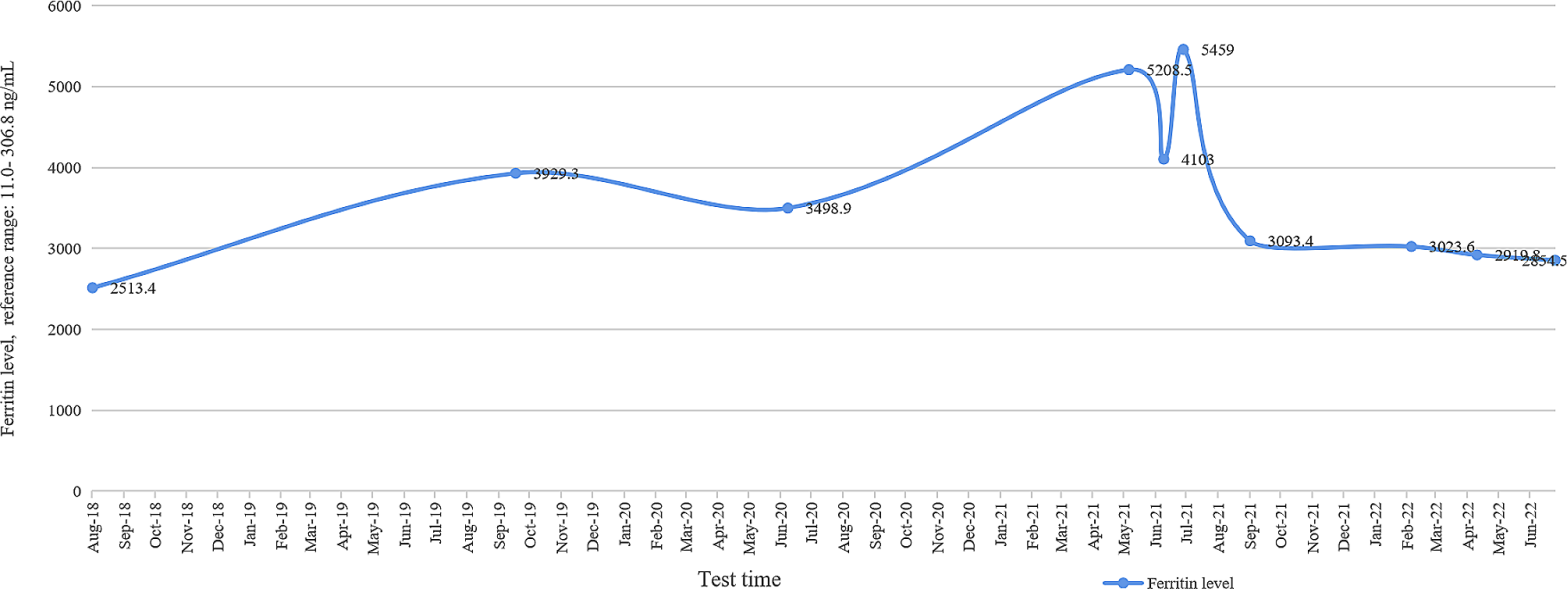
Trend line of the patient’s ferritin level from August 2018 to June 2022

A 31-year-old woman was admitted owing to severe headache and vomiting lasting 1 day in July 2021. The patient had received a blood transfusion 5 days before headache onset. On the morning of admission, she developed an explosive headache, which worsened when sitting or standing, accompanied by dizziness, vomiting, blurred vision, and waist and leg pain. On the day of headache onset, emergency brain computed tomography (CT) showed no obvious abnormalities (Fig. 1A). The patient was previously diagnosed with intermediate α-thalassemia by genetic testing at 2 years of age, after which she received irregular blood transfusions. Transfusion of 2 units of packed RBCs every half a year in 2016, transfusion of 2 units of packed RBCs every 3 months in 2017, and transfusion of 4 units of packed RBCs every 3 months in 2018 were administered owing to anemia. Changes in hemoglobin levels are shown in Fig. 2. A year before admission, liver and cardiac magnetic resonance imaging (MRI) indicated normal cardiac iron levels but revealed severe iron overload in the liver. The patient had been consistently taking deferasirox (1000 mg/day) since 2018, up to the current hospital admission. Changes in ferritin levels are shown in Fig. 3. The patient underwent a splenectomy for thalassemia. She had a history of cholecystectomy, hepatitis, and induced abortion.

Physical examination upon admission showed horizontal nystagmus while looking left, right, and up; negative meningeal signs; and no other abnormal neurological signs. Glycosylated hemoglobin levels were within normal limits, and the results of thyroid and kidney function tests, nucleic acid test for COVID-19, and Coombs test were normal. Abnormal hematological findings are shown in Table 1. Cerebrospinal fluid analysis on day 3 revealed normal pressure, white blood cell count, and glucose and protein levels. Echocardiography showed mild tricuspid regurgitation, chest CT showed atherosclerotic changes in the aorta, and brain MRI (Fig. 1B-H), gadolinium-enhanced MRI (Fig. 1I), MR venography (Fig. 1J), and MR arteriography (Fig. 1K) on day 4 showed no obvious abnormalities. Susceptibility-weighted imaging (Fig. 1L-O) showed abnormal signals in the bilateral and fourth ventricles. Heart and liver iron levels measured by T2-weighted MRI showed severe hepatic iron overload.

**Table 1:** Abnormal findings of blood analysis

Test items	Result	Reference range	
White blood cell counts, ×10^9^/L	14.20	3.5–9.5	Increased
Absolute neutrophils, ×10^9^/L	11.43	1.8–6.3	Increased
Platelet counts, ×10^9^/L	462	125–350	Increased
Mean corpuscular volume, fL	71.9	82–100	Decreased
Total bilirubin, µmol/L	52.5	5.1–19.0	Increased
Direct bilirubin, µmol/L	17.6	0.0–6.8	Increased
Activated partial thromboplastin time, s	44.5	28–44	Increased
Anti-herpes simplex IgG antibodies, signal/ cutoff	10.3	< 1	Increased
Anti-cytomegalovirus antibodies IgG, U/mL	91.3	< 14	Increased
Rubella virus IgG antibodies, IU/mL	26.9	< 10	Increased
Serum total protein, g/L	59.7	63–82	Decreased
Serum albumin, g/L	36.6	40–55	Decreased
Serum creatinine, µmol/L	30	57–97	Decreased
Fibrinogen level, g/L	1.98	2–4	Decreased
Fasting blood glucose, mmol/L	3.8	3.9 ~ 6.1	Decreased
Serum potassium, mmol/L	2.84	3.5–5.3	Decreased

**Fig. 4:**
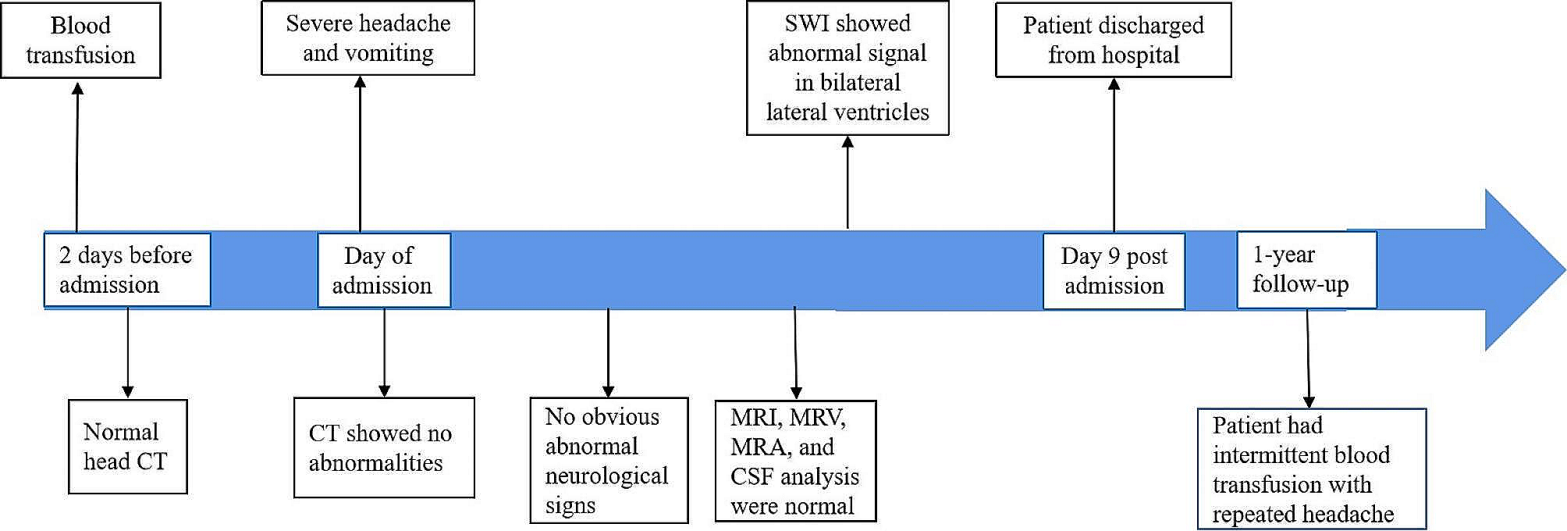
Timeline of the patient’s condition. CSF, cerebrospinal fluid; CT, computed tomography; MRI, magnetic resonance imaging; MRV, magnetic resonance venography; MRA, magnetic resonance arteriography; SWI, susceptibility-weighted imaging

The initial diagnosis, headache, may be secondary to intracranial infection, cerebral venous sinus thrombosis, or reversible cerebral vasoconstriction syndrome (RCVS); therefore, antibiotic (ceftriaxone 2 g/day for 6 days) and antiviral (acyclovir 0.75 g/day for 8 days) treatment, decompression of intracranial pressure (mannitol 375 mL/day), deferasirox 1 g/day, and symptomatic treatment were administered. However, these considerations were not supported by the results of the auxiliary examination. Therefore, transfusion-related headache was considered. The hemoglobin level was 67 g/L on day 3, and the desired transfusion target was a pre-transfusion hemoglobin level of 95–105 g/L (typically to achieve post-transfusion hemoglobin of 130–150 g/L) as per the guidelines [8, 9]. Small intermittent blood transfusions were administered with caution. A RBC suspension (2 units) was transfused on days 4, 5, and 6, and the hemoglobin level was 113 g/L following transfusion. The patient’s headache was gradually relieved, and she was discharged on day 9. Headache recurred after another blood transfusion at the 5-month follow-up; however, it was not as severe as that observed at admission. In the year after discharge, liver and cardiac MRI indicated normal cardiac iron levels but revealed severe iron overload in the liver. The timeline of the patient’s condition is shown in Fig. 4. The patient was satisfied with the treatment and recovered well.

This study was approved by the Ethics Review Board of the First Affiliated Hospital of Shenzhen University (No. 2023-169-01PJ). Informed consent was obtained from the patient.

## Discussion and conclusions

We report a rare case of thalassemia in a patient with CSS who developed severe headache after blood transfusions. After excluding intracranial infection, cerebral venous sinus thrombosis, and RCVS, transfusion-related neurological complications were considered. There is a lack of reports on severe headaches caused by transfusions, particularly when combined with CSS, which can be easily misdiagnosed and lead to over-treatment.

### Pathogenesis of transfusion-related neurological complications

The plasma renin concentration increases after transfusion in patients with thalassemia and returns to normal after a few days [10]. Studies have shown that, in some cases, plasma renin activity and plasma angiotensin II levels started to rise after 6–8 h of transfusion, reached their highest point after 24 h, and then gradually decreased [11]. In conclusion, the evidence suggests the potential for an increase in renin levels following transfusion, although this may be influenced by various factors. Patients with chronic anemia show increased production of endocenesterin-1 (a powerful vasoconstrictor) [12, 13], which may cause arterial spasms and vasoconstriction, leading to headaches. Multiple transfusions of RBCs over a short time can cause changes in blood flow velocity [14], which may lead to headaches. Intermediate and severe thalassemia require long-term blood transfusion and can lead to iron overload, resulting in a hypercoagulable state [2, 15]. The histopathological results in patients with thalassemia who died of cerebrovascular accidents after blood transfusion showed that the findings were similar to those of hypertensive cerebral hemorrhage and hypertensive encephalopathy [6]. In the present case, the patient’s blood pressure was within normal ranges during hospitalization, and hypertensive encephalopathy was not supported.

Blood transfusion may lead to neurological complications, possibly involving immune reactions, electrolyte imbalance, infectious factors, and the characteristics of the blood components themselves [16]. First, during transfusion, the patient may develop antibodies against foreign blood components [17, 18], which may cross-react with structures of the nervous system, leading to inflammation and nerve damage [19]. Second, transfusion may cause electrolyte imbalance, especially changes in potassium and calcium ion concentrations, which may affect the excitability and conduction function of nerve cells. Third, transfusion may spread viruses, bacteria, and other pathogens, which may directly invade the nervous system or indirectly affect neurological function by causing systemic infections. Finally, the large amount of coagulation factors in fresh-frozen plasma may lead to blood clot formation, thereby triggering stroke [20].

### Ferritin and iron deposition

Ferritin levels are closely linked to iron deposition in the brain. Elevated ferritin levels are often associated with increased iron deposition on the brain surface. Conversely, high ferritin levels may indicate or manifest iron deposition on the brain surface. Elevated ferritin levels can lead to iron deposition and damage the brain, liver, and other organs. The spleen is responsible for clearing aging and abnormal RBCs, which release iron as they break down, leading to iron deposition in the spleen. Iron deposits usually appear first in the spleen. Prolonged iron deposition may cause spleen dysfunction, resulting in splenomegaly; this patient underwent splenectomy to treat thalassemia. Pancreatic iron deposition typically occurs in cases of severe imbalance in iron metabolism. The liver is the main organ for iron metabolism and storage. Iron deposition in the liver usually progresses more slowly than in the spleen and pancreas, but once it begins, the accumulation rate may accelerate. Cardiac iron deposition may occur in the middle to late stages of the disease, leading to myocardial damage and heart failure. The timing of iron deposition in the brain is not fully understood, but it may damage nerve cells and lead to decreased cognitive function.

### Clinical characteristics of transfusion-related neurological complications

Thalassemia patients experienced a higher prevalence of headaches compared to the general population [21]. Headaches may result from insufficient oxygen supply to the brain [22]. The body responds to the lack of oxygen due to anemia by raising heart rate and blood flow speed. This compensatory mechanism can cause the dilation of cerebral blood vessels, leading to headaches. Severe headache after blood transfusion is rarely reported, and this aspect is not fully understood. In 1979, Wasi et al. first reported that eight patients with thalassemia developed hypertension, convulsions, severe headache, and cerebral hemorrhage after transfusion of 3–7 units of blood; the authors emphasized that this was a unique clinical syndrome caused by blood transfusion [5]. CNS complications (e.g., convulsions [5] and disturbance of consciousness [23]) may occur in patients with thalassemia after transfusions [6, 23]. Some transfusion-related headaches are secondary to cerebral hemorrhage, but severe headaches alone are also one of the complications of transfusion.

The characteristics of transfusion-related headache alone in patients with thalassemia are as follows: (1) severe headaches are the most common, while hypertension, loss of consciousness, convulsions, cerebral hemorrhage, etc., may not appear; (2) headaches usually occur 2–15 days after the last transfusion and often occur after multiple transfusions within a short time, as shown in this case report. The onset of headache may not be accompanied by an obvious neurological disease. To the best of our knowledge, there are few reports of headaches caused by transfusion in other diseases, and transfusion-related headaches may be relatively specific to patients with thalassemia.

### Differential diagnosis of transfusion-related headaches

The patient in this study exhibited unique signs of CSS, and it was crucial to determine whether the patient’s headache was related to CSS. CSS is caused by the deposition of hemosiderin and iron ions on the surface of the CNS, and its clinical features include binaural sensorineural hearing loss, progressive cerebellar ataxia, and pyramidal tract sign [24]. CSS deposits are mainly located under the pia mater and on the surface of the brain tissue, cranial nerves, and spinal cord [25, 26]. CSS is characterized by the deposition of hemosiderin in the pial and subpial regions of the brain, resulting from chronic bleeding in the superficial layers of the cortex and spinal cord [27]. The etiologies of subarachnoid bleeding leading to CSS are broad and heterogeneous, including tumors, vascular malformations [28], nerve root avulsions, and iatrogenic [29], traumatic, and pathological dural causes, as well as cerebral amyloid angiopathy [30]. In this case, CSS may have been caused by long-term chronic hemolysis, and repeated blood transfusions may have led to iron overload [31, 32]. CSS can lead to a spectrum of neurological deficits and symptoms, including transient focal neurological episodes, mild cognitive impairment, dementia, and stroke [33, 34]. The condition can result in sensorineural hearing loss, ataxia, pyramidal signs, and vestibular deficits, which are characteristic of CSS [35]. There was a strong temporal correlation between the headache and blood transfusion; therefore, we considered headache as a possible CNS complication associated with blood transfusion.

RCVS must also be ruled out, which mainly presents with reversible, sudden thunderclap pain with or without neurological deficits and diffuse cerebral vasoconstriction that can recover within 3 months [1, 36]. Exercise, swimming, sexual intercourse, dysthymia, stress, and cough are common predisposing factors of RCVS, and RCVS is common in people who use vasoactive drugs and during postpartum [37]. There were no obvious abnormalities on MR arteriography during headache in the present case; therefore, a diagnosis of RCVS was not supported. Intracranial infection was excluded based on clinical information including signs and results of lumbar puncture.

### Treatment of transfusion-related headaches

Symptomatic treatment is the main treatment for the acute phase of headache in such patients, and there is currently a lack of specific treatment. Iron chelation therapy helps reduce the damage caused by the deposition of iron in organs such as the brain and liver. In severe cases, higher dosages of deferasirox, typically ranging from 20 to 40 mg/kg/day, can be considered, depending on the amount of transfusional iron loading, even in children [38]. Alternatively, critically ill patients can be administered intravenous deferoxamine at an initial dose of 1000 mg, followed by 500 mg every 4–12 h [39]. It is essential to gradually maximize the dosage of these iron chelators in severe cases to achieve optimal therapeutic outcomes while minimizing adverse effects. In certain clinical situations, the use of hydroxyurea can increase HbF levels and improve anemia [40, 41]. Supplementing with antioxidants and magnesium may help alleviate post-transfusion vasoconstriction and secondary headaches [42, 43]. Timely identification of transfusion-related headaches can help minimize unnecessary tests and treatments. Formulation of a more scientific and reasonable blood transfusion protocol may help reduce transfusion-related headaches in patients with thalassemia.

### Limitations

The mechanisms of severe headache in patients with thalassemia after blood transfusion may be related to a variety of factors, and blood transfusion is an important trigger; however, the exact mechanism remains unclear. Further research will help prevent and treat these serious complications.

## Conclusions

Post-transfusion headache in patients with thalassemia has not been fully recognized and is easily misdiagnosed, leading to excessive examination and treatment. Understanding the clinical features of transfusion-related headaches can help identify these complications. However, the exact mechanism requires further research to help prevent and treat this serious complication.

## Data Availability

All data generated or analyzed during this study are included in this published article.

## Abbreviations

CNS: Central nervous system
CSS: Cerebral superficial siderosis
CT: Computed tomography
MR: Magnetic resonance
MRI: Magnetic resonance imaging
RBC: Red blood cell
RCVS: Reversible cerebral vasoconstriction syndrome
